# Supplementary material for: Using a mobile health app to improve patients’ adherence to hypertension treatment: a non-randomized clinical trial
Source: PeerJ. 2021 May 28;9:e11491. doi: 10.7717/peerj.11491 (PMC8166239; doi:10.7717/peerj.11491)
Supplement: Supplemental Information 2 [file peerj-09-11491-s002.pdf]

Protocol

An Electronic Health Platform for Monitoring Health Conditions of Patients With Hypertension in the Brazilian Public Health System: Protocol for a Nonrandomized Controlled Trial

Ana Carolina Bertoletti De Marchi[1,2,3], BSc, MSc, PhD; Ana Luisa Sant' Anna Alves[2], MSc, PhD; Carla Beatrice Crivellaro Gonçalves[4], BPharm, MSc, PhD; Cristiano Roberto Cervi[1,3], BSc, MSc, PhD; Daiana Biduski[1], BSc, MSc; Ericles Andrei Bellei[1], BTech, MSc; Guilherme Afonso Madalozzo[1,3], BSc, MSc, PhD; Ivana Beatrice Mânica Da Cruz[5], BSc, MSc, PhD; Jeangrei Emanoelli Veiga[1,3], BTech, MSc; João Pedro Mazuco Rodriguez[3], BSc; Luciano Rodrigo Ferretto[1], BSc, MSc; Luiz Carlos Pereira Bin[6], MD; Marcelo Trindade Rebonatto[1,3], BSc, MSc, PhD; Marilene Rodrigues Portella[2], MSc, PhD; Mateus Klein Roman[3], BSc; Nathália Pinto Cechetti[1], BSc, MSc; Rafael Rieder[1,3], BSc, MSc, PhD; Raquel Debon[2], MSc; Simiane Salete Volpi[2]

[1]Graduate Program in Applied Computing, Institute of Exact Sciences and Geosciences, University of Passo Fundo, Passo Fundo, Brazil
[2]Graduate Program in Human Aging, College of Physical Education and Physiotherapy, University of Passo Fundo, Passo Fundo, Brazil
[3]Undergraduate Program in Computer Science, Institute of Exact Sciences and Geosciences, University of Passo Fundo, Passo Fundo, Brazil
[4]Municipal Health Department, Passo Fundo, Brazil
[5]Graduate Program in Gerontology, Center of Physical Education and Sports, Federal University of Santa Maria, Santa Maria, Brazil
[6]Hospital of Clinics of Passo Fundo, Passo Fundo, Brazil

Corresponding Author:
Ana Carolina Bertoletti De Marchi, BSc, MSc, PhD
Graduate Program in Applied Computing
Institute of Exact Sciences and Geosciences
University of Passo Fundo
B5 Building, São José
Highway BR285, Km 292
Passo Fundo, 99052900
Brazil
Phone: 55 54 33168354
Email: carolina@upf.br

Abstract

Background: Chronic noncommunicable diseases such as arterial hypertension have a high impact in the context of public health. Previous studies have shown improvements in blood pressure due to simple lifestyle changes, which were supported by electronic health (eHealth) solutions.

Objective: The aim of this study is to develop an eHealth platform and assess the effects of its use on the health conditions of patients with hypertension, with assistance from health professionals in the public health system of a Brazilian city.

Methods: The platform will include a server that centralizes all the data and business rules, a website dashboard for health professionals, and a mobile app for patients. We will analyze the effects of its use through a controlled, nonrandomized, nonblind, prospective, monocentric clinical trial. We will enroll 68 participants diagnosed with arterial hypertension and under medical follow-up and categorize them into two groups. The participants of the intervention group will use the platform as a monitoring method, whereas the participants of the control group will use conventional methods. In both groups, we will assess and compare the evolution of blood pressure and treatment adherence before, during, and after the intervention.

Results: The project was funded at the end of 2018. We have been developing the software since 2019 with plans to complete it in 2020, and we will enroll patients between 2020 and 2021. We expect to submit the first results for publication in 2020.

Conclusions: For the primary outcome, we expect a reduction and stabilization of blood pressure. For the secondary outcomes, we hope to see improvements in treatment adherence, physical activities and dietary practices, and acceptance of the eHealth

XSL•FO
RenderX

platform. In public health, the technology that favors disease control also helps reduce complications and, consequently, treatment costs. The platform might encourage the adaptation of medical assistance to incorporate this technology into patient monitoring.

International Registered Report Identifier (IRRID): PRR1-10.2196/15299

(JMIR Res Protoc 2020;9(1):e15299) doi: 10.2196/15299

KEYWORDS

hypertension; eHealth; mHealth; public health; Brazil

Introduction

Background

Chronic noncommunicable diseases such as arterial hypertension are responsible for a high frequency of hospitalizations and have a great socioeconomic impact [1]. In Brazil, the direct and indirect costs of these diseases have been increasing significantly [2]. Currently, 32% of the Brazilian adult population, which corresponds to 36 million individuals, has hypertension [3]. However, only about half of the patients under treatment for hypertension report that their blood pressure is within the recommended levels [3,4]. As hypertension is a chronic condition, the best approaches for a healthy life are the awareness, treatment, and management of the disease.

With this perspective, the Brazilian Ministry of Health developed a system called Brazilian Clinical Management System of Arterial Hypertension and Diabetes Mellitus (SIS-Hiperdia) for registering and monitoring patients with arterial hypertension and diabetes mellitus, with assistance from the health professionals in the outpatient clinics of the Brazilian public health network. However, patients’ data are required for continued working of this system, and it is not feasible to obtain this information if patients do not have a history of their health conditions available [5]. Moreover, a strategy developed for use only in clinical centers may not be enough to treat the disease and change habits. Developing and using complementary tools is necessary for both physicians and patients [6]. Therefore, the electronic health (eHealth) systems have been attracting an increasing number of users because of the diffusion of mobile devices and the growing interest in better quality of life and health care [7]. The World Health Organization [8] announced that eHealth solutions might transform the resources of health services worldwide. Most regions in the world, including low- and medium-income countries, are working actively on eHealth projects [8], including Brazil.

Previous studies have shown positive results in the improvement of blood pressure from simple lifestyle changes supported by eHealth solutions [9]. The results by Toro-Ramos et al [10] indicated a decrease in diastolic pressure and body weight of the 73 participants. In the study by Ashoorkhani et al [11], tests for 132 patients with hypertension showed an improvement in treatment adherence, weight control, and regular blood pressure verification. The study by Albini et al [12] with 690 hypertensive patients suggests that information and communication technology tools may be effective in providing physicians with dynamic patient control and in improving the monitoring of hypertension and adherence to treatment prescriptions.

In the studies mentioned above [10-12], periodic verification was essential for obtaining favorable results. However, a prominent challenge in the market of mobile apps is maintaining long-term or recurrent use of the app among users engaged with the app, without loss of interest. Among the leading causes of abandonment from the users of these apps are quitting after achieving goals and the lack of resources available [13]. Several techniques have been used to allow greater user engagement with the app, such as gamification, which comprises applying gaming elements to a nongaming context [14].

Prior Work

We have devised a platform for monitoring the health conditions of patients with arterial hypertension. We implemented an initial version, considered a prototype, and made it available to use for initial validations. The platform includes a server that centralizes the data for a website interface [15] that is designed for health professionals, and a mobile app with gamification elements [16] that is designed for patients.

We performed pilot studies with health professionals and patients to assess the prototype functionalities [15] and patient engagement and experience with the app [16-18], respectively. The assessment with health professionals showed some benefits of using the system, such as the optimal visualization of patients’ logbooks. Patient assessments showed that gamification favors engagement with health treatment. We also found that participants under follow-up with a health professional interacted more with the app and were more motivated to maintain their health monitoring. Therefore, the use of digital health resources by professionals, along with patients, may promote a higher commitment to monitoring the treatment factors [17]. To verify the clinical effects, we have already conducted a pilot trial [19], including 39 patients with arterial hypertension, to compare the use of the platform’s initial version with conventional hypertension monitoring over a period of 3 months. The patients who used the mobile health app had a change in systolic and diastolic pressure toward more adequate levels. In addition, the group had improved levels of glucose and high-density lipoprotein cholesterol and a reduced consumption of ultraprocessed foods.

The main goal of our project is to assess the effects of using the eHealth platform—which will be developed—on health conditions of patients with hypertension, with assistance from health professionals in the public health system.

XSL•FO
RenderX

Methods

Notice MS-SCTIE-Decit CNPq 12/2018: Research on Health Innovation

Our eHealth platform potentially favors the public health system by allowing the monitoring of health conditions of patients with hypertension, assessing risk, and supporting behavior changes. However, as it is an initial version, the software still requires improvement and the development of other functionalities and resources. The new characteristics predicted include a nutritional assessment model, a recommendation system, and the integration with wearable devices equipped with physiological sensors to automate data collection.

After a strict selection process of Notice MS-SCTIE-Decit CNPq 12/2018, the Brazilian Ministry of Health and the Brazilian Counsel of Technological and Scientific Development granted our project with research funding. Considering the potential for health innovation, they selected our eHealth platform for full development and testing in the Brazilian public health system.

Software Specification

The eHealth platform (Figure 1) is a solution that aims to monitor patients with hypertension by integrating risk assessment and counseling for healthy habit changes. This occurs with a joint action among resources for registering health conditions and receiving notifications, reminders, and follow-up from health professionals. The platform includes a server that centralizes all data and business rules; a website dashboard for health professionals; and a mobile app for patients, which is integrated with a wearable device. The Brazilian Institute of Industrial Property granted us the registration of the first versions of the website dashboard and the mobile app (documents BR5120170007616 and BR5120180009735, respectively).

Figure 1. Computing architecture of the electronic health platform.

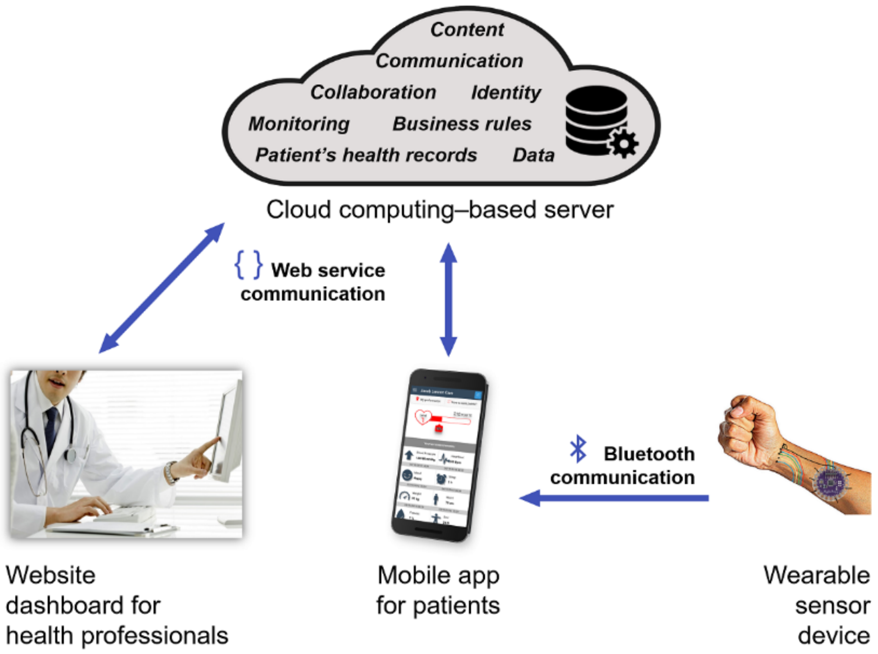

Website Dashboard

We designed the dashboard for health professionals, so that they could follow-up and monitor patients in real time. The professionals have access to patient information, and they may assess risks and advise patients remotely. In addition, the dashboard has an interface for the administrator to control business rules and data processing, with settings related to the frequency of alerts, monitoring the schedule of patient reminders, user access control, clinical guide parameters (eg, blood pressure, body fat, and body mass index), and messaging, among others.

After registration and access, the system forwards the professional to the functionalities of messages and alerts on patient measurements. To start the monitoring of a patient, professionals need to link the patient profile to their account. After patient authorization, the professional will have access to the patient's logbook of blood pressure, heartbeat, weight, mood, waist circumference, body fat, sleep, physical activity, schedule, and dietary intake assessment. The platform can display all the data in graphic, interactive, and associated views.

More than one health professional may assist the patient, for instance, a cardiologist, a dietitian, and a psychologist. Thus, each professional may choose the information they wish to supervise according to each patient and the patient's specific treatment. When selecting an item, the professionals proceed to a screen containing detailed information of the patient's history to select what period they want to visualize. They may also view the reminders included by the patient, enter new reminders, and communicate with the patient through a private chat.

The study by Veiga [15] presented all the details of the functional prototype. The dashboard prototype will be the basis

XSL•FO
RenderX

for the full development of the new version, which will also include new functionalities such as modules for nutritional assessment and recommendation of physical exercises and dietary habits for each patient profile, as developed by Ferreto [20].

Mobile App

The app allows users to record their data, send and receive notifications, communicate with their medical team, and pair the wearable device that will be developed. After installation and first registration, the patient can access the main interface containing his or her last measurements. Through the main interface, users can access the menu with the list of functionalities available, which include height, physical activity, heartbeat, waist, body fat, mood, weight, blood pressure, sleep, reminders, and chat. In the new version, other records will be available, including dietary intake and improvement of physical activity data. When selecting a functionality, patients can view their record information and graphs, and they may include, change, or delete data.

For the measurements, the patient records the date, time, value, and an optional observation. All operations with patient data synchronize with the central server. Periodically, the app displays alert notifications and reminders on the health data recorded, including notes sent by the health professional. Each notification presents a title, a brief description, and buttons with *yes* and *no* options. When clicking *yes*, the system sends the information to the server, showing whether the patient completed the action or reminder presented, for instance, whether they took the medication or visualized the message from the health professional. Such integration aims at continuous monitoring and the stimulus to maintain healthy practices.

We designed all functionalities of the app to engage patients with a regular health follow-up. In addition to the gamification elements, they also receive reminders and notifications on their health and interact with health professionals. The app has a functionality responsible for notifying the patient and the professional about health evolution through counseling based on clinical guidelines. At each insertion of patient data, the system analyzes the information according to the guidelines and issues an alert notification if the measurement is identified as different from the standard patterns. On the basis of these notifications, the patient may analyze and decide what to do next, including using the messaging functionality to contact the health professional for assistance. This integration does not replace personal visits, but it provides both the patient and professional with a fast and accessible communication tool for doubt resolution and guidance. All the records create a lifestyle logbook of the patient under monitoring, which may represent a source of information for the decision-making process of professionals.

The study by Cechetti et al [17] presented all the details of the functional prototype. The app prototype will be the basis for the full development of the new version, which will also include new functionalities corresponding to the ones predicted for the website dashboard.

Wearable Sensor Device

We will build a device equipped with modules of physiological sensors available in the market, such as oximeter and pedometer, compatible with the LilyPad Arduino—a mini-controller board designed for wearables and intelligent textiles. The device will have a communication routine with the mobile app through the Bluetooth protocol to automate the measurement records.

First, we will assess the use of the wearable device in a group of 10 individuals. We will connect the device to a piece of clothing (eg, T-shirt, wristband, and glove) worn by the participant to monitor the physiological signs during daily activities such as walking, lying down, and nonexhaustive work. On the basis of a study by Zhou et al [21], we prepared a questionnaire to assess the level of comfort while using the device. If we detect any nuisance in the comfort assessment, we will adjust the device to suppress such discomfort.

We will perform technical trials to test the correct functioning of the equipment and wireless communication assessments with performance measures (eg, response time, package traffic, and latency) and the request acknowledgment and checksum. We will also evaluate the behavior of electronic components attached to the hardware modules to ensure the reliability and accuracy of the collected data. For this validation, we will compare values obtained by the sensors with values recorded by reliable conventional devices used by health professionals, such as sphygmomanometer, heart monitor, and pulse oximeter. We will perform all assessments of the wearable sensor device separately in substudies, which will not be a part of the main clinical trial.

Nonrandomized Controlled Trial

This is an applied research of technological innovation with software development and analysis of its effects through a controlled, nonrandomized, nonblind, prospective, and monocentric clinical trial. The Research Ethics Committee of the University of Passo Fundo, Rio Grande do Sul, Brazil (report 3.414.793) approved this project, which was registered in the Brazilian Platform of Clinical Trials under the code RBR-2rkkgn (Universal Trial Number U1111-1230-7550). Figure 2 presents the trial design and flowchart.

XSL•FO
RenderX

Figure 2. Trial procedure flowchart. eHealth: electronic health; MBG: Martín-Bayarre-Grau; SUS: System Usability Scale; TAM: technology acceptance model.

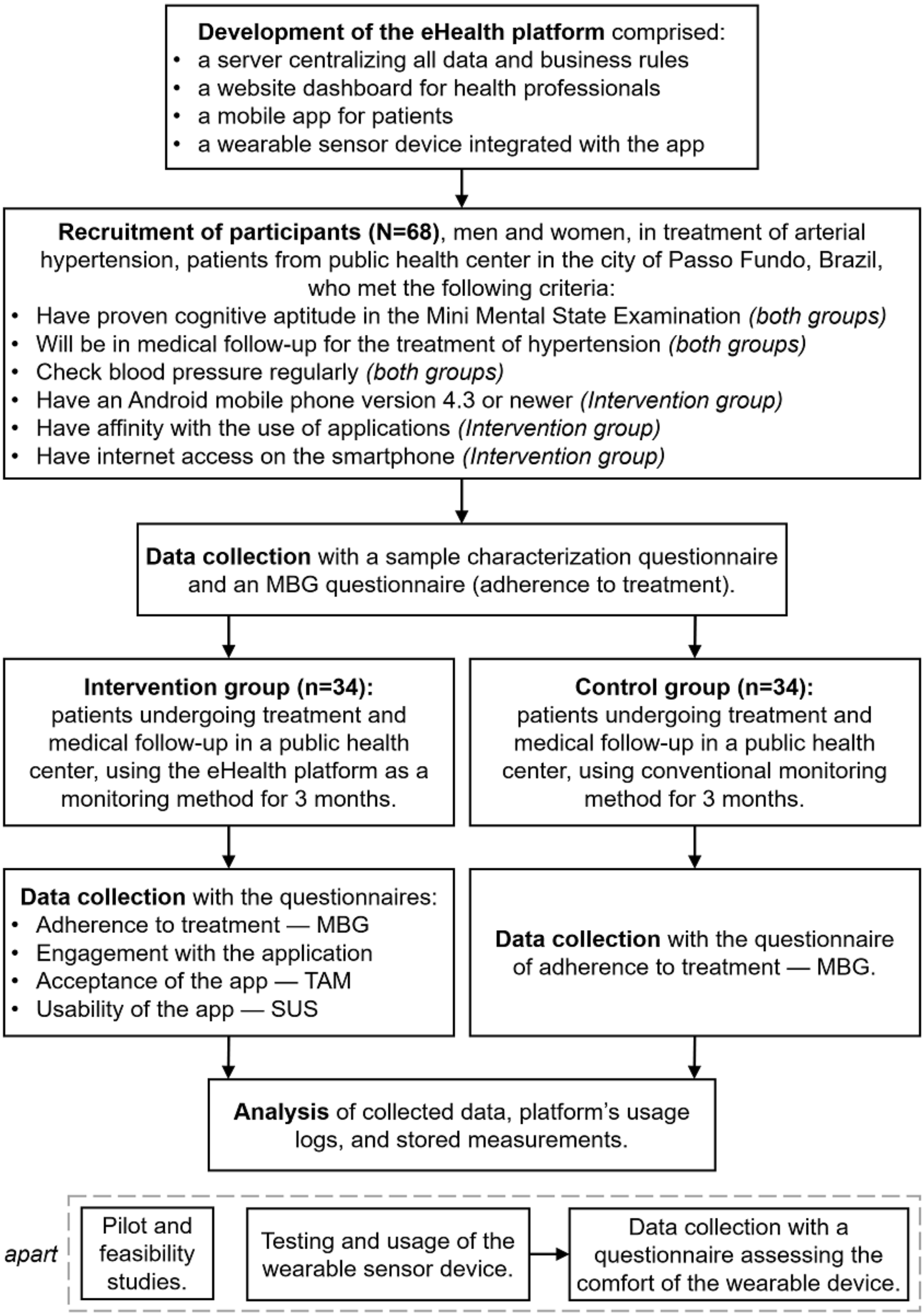

Study Population and Sample

The study population will include men and women under treatment for arterial hypertension who will be assisted by health professionals in the public health system of a Brazilian city. After the invitation to take part in the study, the participants will sign a consent form. The effect (primary outcome) is expected to be 80% in the intervention group and 40% in the control group, which means a 40% difference. These proportions are based on the findings of the study by Neumann et al [22], which was conducted with a similar design, and also on more optimistic results obtained from one of our pilots [19]. For sample calculation, we used Epi Info version 6.0 software (Centers for Disease Control and Prevention, Atlanta, Georgia; sample size/2 proportions). We considered an estimate of 40% proportion difference between groups, 95% confidence interval,

XSL•FO
RenderX

and 80% statistical power (beta). The calculation resulted in 28 individuals for each group, but to minimize the effects of loss and refusal during the intervention, we added 20% to the size, yielding a total of 34 individuals in each group.

The allocation of patients in each group will be based on the eligibility criteria. For example, if the patient fits all the criteria but does not have a mobile phone compatible with the app, then he or she will be allocated to the control group. If the patient has a mobile phone compatible with the app, he or she will be allocated to the intervention group. For propensity score calculation, based on the effect of confounding factors or predictors of exposure (to intervention), we will consider demographic variables of age, gender, and socioeconomic status.

We will consider the following inclusion criteria:

- Cognitive ability confirmed by the Mini-Mental State Examination [23] (both groups)
- Current medical monitoring for the treatment of hypertension in the public health system of the city of Passo Fundo, RS, Brazil (both groups)
- Possibility to measure blood pressure in drugstores, hospitals, Family Health Strategies, or centers for comprehensive health care (both groups)
- Possession of an Android mobile phone, version 4.3 or higher (intervention group)
- Familiarity with the use of apps (intervention group)
- Internet access on the smartphone (intervention group).

Evaluation and Assessment Frameworks

We will use the following assessment instruments:

- Mini-Mental State Examination: It verifies the integrity of cognitive functions, assessing the functions of space-time orientation, memory, attention, calculus, language, and constructive praxis. It includes 11 tasks, with a score ranging from 0 to 30 points. Brucki et al [23] adapted it to the Brazilian context. The cut-off points relate to the years of schooling of the subjects, ie, for illiterate individuals, 19 points; 1-3 years of schooling, 23 points; 4-7 years of schooling, 24 points; and more than 7 years of schooling, 28 points.
- Sample characterization and sociodemographic questionnaire: It includes questions on age, level of education, income, and experience in using a blood pressure meter. The project researchers will provide the specifications.
- Martín-Bayarre-Grau questionnaire (MBG): It assesses the therapeutic adherence for arterial hypertension. Alfonso et al [24] developed and validated this instrument, and Matta et al [25] adapted it to the Brazilian Portuguese language.
- Dietary intake questionnaire by the Brazilian Food and Nutrition Surveillance System (SISVAN): from the Brazilian Ministry of Health [26], this questionnaire identifies the intake frequency of some foods and beverages over the last 7 days through 10 questions. Each item may relate to healthy food or nonrecommended unhealthy food.
- Engagement questionnaire: It assesses the attributes of engagement, such as esthetics, sensory appeal, concentration, awareness, challenge, control, feedback, interest, motivation, novelty, and time perception. O'Brien and Toms [27] proposed and validated the model, and Cechetti et al [17] translated it to Portuguese.
- Acceptance questionnaire: This is based on the technology acceptance model (TAM) [28]. The assessment relies on three categories: perceived utility (determines the degree to which a person believes the use of a technology may improve performance and productivity), ease of use (determines the degree to which a person believes the use of a system will be easy to learn and interact with), and external variables (provide understanding about what influences perceived utility and ease of use).
- Usability assessment questionnaire: This is in accordance with the System Usability Scale (SUS), related to the ease of use and use effort. Brooke [29] developed this framework.
- Comfort assessment questionnaire for the wearable device: It aims to verify the comfort and well-being of users while trying the wearable technology. The project researchers will produce this instrument.
- Characteristics of app use: It considers the number of accesses, number of records per period, response to notifications, and reminders. The system usage logs will calculate all this information. We will also use open questions so that participants can report their experience.
- Health professional assessment: We will perform a semistructured interview with health professionals to verify opinions on the characteristics and resources of the eHealth platform. Questions include, but are not limited to the following: (1) As a health professional, do you believe that the app and dashboard's features can facilitate the clinical practice to monitor a patient? In what way? (2) Do you believe that a patient can benefit from the app's features in his or her treatment? In what way? (3) What changes do you suggest for this platform to make it more useful or applicable to clinical practice?

Procedure

We will collect data at three timepoints: preintervention, during the intervention, and postintervention. We will develop the wearable device and assess it in parallel outside the clinical trial.

Preintervention Period

After verifying the inclusion criteria, all participants will have a 1-hour meeting with the researchers for instructions on the objective of the study and for signing a consent form detailing all the study procedures. We will register the participants of the intervention group in the system and instruct them about the app's use. We will collect the preintervention data from both groups from a sample characterization questionnaire and an MBG questionnaire [24].

Intervention Period

Participants from the control group will perform conventional hypertension monitoring using paper logbooks. Participants from the intervention group will use the app for 3 months, periodically registering the values of blood pressure, weight, height, sleep quality, waist circumference, mood, and heartbeats,

XSL•FO
RenderX

among others. Health professionals from the health centers will assist the participants by using the website dashboard to monitor and interact with their patients. Professionals' performance will not be evaluated, but we will ask them to participate by communicating with patients weekly on the platform. We will use all the data recorded by patients and health professionals during the intervention in the final analysis of results, especially the measurements of blood pressure and frequency of physical activity.

Postintervention Period

All participants will have a 1-hour meeting with the researchers for the final data collection. We will collect the postintervention data with an MBG questionnaire [24], an engagement questionnaire [27], an acceptance questionnaire (TAM) [28], and a usability questionnaire (SUS) [29].

Statistical and Qualitative Analysis

Our expectation for the primary outcome is the reduction and stabilization of blood pressure. For the secondary outcome, we hope for acceptance of the eHealth platform and improvements in treatment adherence, physical activities, and dietary intake. A priori levels will be set according to the assessment tool and the expected outcome. The acceptance of the eHealth platform will consider good scores obtained in the analysis of usage logs and questionnaires of engagement, SUS, and TAM. Improved adherence to treatment will consider MBG questionnaire scores measured before and after the intervention. For the analysis of physical activities and dietary intake, a binary outcome will consider whether the patient has adequate levels, diagnosed according to the SISVAN's questionnaire and standards.

For qualitative data, we will use Minayo's guidelines [30]. Numeric data will be analyzed with statistical software SPSS 22 (IBM Corp, Armonk, New York). We will calculate the measures of central tendency and dispersion of the quantitative variables, and we will use the Kolmogorov-Smirnov test to verify normality. Basic quantitative data will be determined by mean, standard deviation, and median. Categorical data will be determined by simple frequency. Blood pressure data will be analyzed with the paired *t* test. Either the paired *t* test (parametric) or the Wilcoxon test (nonparametric) may be applied to compare other quantitative variables before and after the intervention. To assess treatment adherence, physical activity, and dietary intake after intervention, we will apply the Chi-square test while considering the app use as the dependent variable; the independent variables will be treatment adherence, physical activity, and dietary intake. For the MBG questionnaire, we will apply the Mann-Whitney test to compare the score between the groups before and after the intervention. The same test may be used to compare diastolic and systolic blood pressure measurements between the preintervention and postintervention groups. We will consider a 95% confidence interval ($P<.05$).

To evaluate the findings' heterogeneity (primary and secondary outcomes), we will apply a statistical technique to verify whether the differences observed in the results can be explained by chance. We will apply the Chi-square test with a significance level of $P<.10$. The magnitude of heterogeneity will be assessed by calculating the I^2 value, which ranges from 0% to 100%. An I^2 greater than 50% indicates substantial heterogeneity, and an I^2 above 75% indicates considerable heterogeneity. For the evaluation of heterogeneity, we will consider the demographic and socioeconomic variables. In the presence of heterogeneity, we will investigate its causes through sensitivity analysis. In the sensitivity analysis, the form of data analysis varies, to identify the impact of this change on the results. All analysis techniques can be adjusted or modified, if required, by the characteristics of the collected data. If feasible, we also intend to analyze the results with logistic regression of preintervention and postintervention data. In this case, the dependent variable will consider the base category *whether* [user] *used the app*, related to independent, categorized, and continuous variables of interest such as sociodemographic variables and the expected secondary outcomes.

Results

The project was funded at the end of 2018. We will develop the software between 2019 and 2020, and we will enroll patients between 2020 and 2021. We expect to submit the first results for publication in 2020. As the final technological product, we will offer an innovative and technological eHealth platform to the public sector. The transfer of technology from the academic environment to the public sector is a strategy to invigorate efforts and enable improved health conditions to the population.

Discussion

The expected results may contribute to improving the quality of life of patients with arterial hypertension. Using eHealth technology will allow a better understanding of the importance of permanent health care. Furthermore, it will provide encouragement for health monitoring and risk assessment, favoring behavior changes toward healthier habits [31-33]. In public and primary health, the technology that favors disease control also helps reduce complications and, consequently, treatment costs [34,35]. The eHealth platform encourages the adaptation of medical assistance to facilitate the incorporation of technology into patient monitoring, especially for those living in rural areas or regions of difficult access [36,37]. Nevertheless, introducing the use of technology may simplify health information records and communication between patients and professionals [38]. The health database created may further benefit and integrate Brazilian systems such as the SIS-Hiperdia.

Acknowledgments

The National Council of Scientific and Technological Development; the Brazilian Ministry of Health; and the Brazilian Ministry of Science, Technology, Innovation, and Communication are funding this project through grant number 440078/2018-0. The authors are solely responsible for designing and performing this study and all its analyses, for drafting and editing the manuscript, and for the final contents.

XSL•FO
RenderX

Conflicts of Interest

None declared.

Multimedia Appendix 1

CONSORT-EHEALTH checklist (V 1.6.1).
[PDF File (Adobe PDF File), 2316 KB-Multimedia Appendix 1]

References

1. Malachias M, Plavnik FL, Machado CA, Malta D, Scala LC, Fuchs S. 7th Brazilian guideline of arterial hypertension: chapter 1 - concept, epidemiology and primary prevention. Arq Bras Cardiol 2016 Sep;107(3 Suppl 3):1-6 [FREE Full text] [doi: 10.5935/abc.20160151] [Medline: 27819380]
2. Siqueira AD, Siqueira-Filho AD, Land M. Analysis of the economic impact of cardiovascular diseases in the last five years in Brazil. Arq Bras Cardiol 2017 Jul;109(1):39-46 [FREE Full text] [doi: 10.5935/abc.20170068] [Medline: 28591251]
3. Gandra A. Brazil Agency. 2018 May 17. Brazil Participates in World Database on Hypertension URL: http://agenciabrasil.ebc.com.br/saude/noticia/2018-05/brasil-participa-de-banco-de-dados-mundial-sobre-hipertensao [accessed 2019-11-11]
4. Vilaplana J, Solsona F, Abella F, Cuadrado J, Teixidó I, Mateo J, et al. H-PC: a cloud computing tool for supervising hypertensive patients. J Supercomput 2015;71(2):591-612. [doi: 10.1007/s11227-014-1312-9]
5. Bellei EA, Biduski D, Cechetti NP, de Marchi AC. Diabetes Mellitus m-Health applications: a systematic review of features and fundamentals. Telemed J E Health 2018 Nov;24(11):839-852. [doi: 10.1089/tmj.2017.0230] [Medline: 29470105]
6. Bellei EA, Biduski D, Lisboa HR, De Marchi AC. Development and assessment of a mobile health application for monitoring the linkage among treatment factors of Type 1 Diabetes Mellitus. Telemed J E Health 2019 Feb 6. [doi: 10.1089/tmj.2018.0329] [Medline: 30724717]
7. Eysenbach G. What is e-health? J Med Internet Res 2001;3(2):e20 [FREE Full text] [doi: 10.2196/jmir.3.2.e20] [Medline: 11720962]
8. World Health Organization. mHealth: New Horizons for Health through Mobile Technologies. Geneva: World Health Organization; 2011.
9. Debon R, Coleone JD, Bellei EA, De Marchi AC. Mobile health applications for chronic diseases: a systematic review of features for lifestyle improvement. Diabetes Metab Syndr 2019;13(4):2507-2512. [doi: 10.1016/j.dsx.2019.07.016] [Medline: 31405669]
10. Toro-Ramos T, Lee D, Kim Y, Michaelides A, Oh TJ, Kim KM, et al. Effectiveness of a smartphone application for the management of metabolic syndrome components focusing on weight loss: a preliminary study. Metab Syndr Relat Disord 2017 Nov;15(9):465-473. [doi: 10.1089/met.2017.0062] [Medline: 29035677]
11. Ashoorkhani M, Bozorgi A, Majdzadeh R, Hosseini H, Yoonessi A, Ramezankhani A, et al. Comparing the effectiveness of the BPMAP (Blood Pressure Management Application) and usual care in self-management of primary hypertension and adherence to treatment in patients aged 30-60 years: study protocol for a randomized controlled trial. Trials 2016 Oct 21;17(1):511 [FREE Full text] [doi: 10.1186/s13063-016-1638-0] [Medline: 27769282]
12. Albini F, Liu X, Torlasco C, Soranna D, Faini A, Ciminaghi R, et al. An ICT and mobile health integrated approach to optimize patients' education on hypertension and its management by physicians: The Patients Optimal Strategy of Treatment(POST) pilot study. In: Proceedings of the 2016 38th Annual International Conference of the IEEE Engineering in Medicine and Biology Society. 2016 Presented at: EMBC'16; August 16-20, 2016; Orlando, FL, USA p. 517-520. [doi: 10.1109/EMBC.2016.7590753]
13. Murnane EL, Huffaker D, Kossinets G. Mobile Health Apps: Adoption, Adherence, and Abandonment. In: Adjunct Proceedings of the 2015 ACM International Joint Conference on Pervasive and Ubiquitous Computing and Proceedings of the 2015 ACM International Symposium on Wearable Computers. 2015 Presented at: UbiComp/ISWC'15 Adjunct; September 7 - 11, 2015; Osaka, Japan p. 261-264. [doi: 10.1145/2800835.2800943]
14. Deterding S, Dixon D, Khaled R, Nacke L. From Game Design Elements to Gamefulness: Defining 'Gamification'. In: Proceedings of the 15th International Academic MindTrek Conference: Envisioning Future Media Environments. 2011 Presented at: MindTrek'11; September 28 - 30, 2011; Tampere, Finland p. 9-15. [doi: 10.1145/2181037.2181040]
15. Veiga JE. TEDE UPF. 2017. An E-Health System to Assist in the Promotion of Healthy Lifestyle in Hypertension URL: http://tede.upf.br/jspui/handle/tede/37 [accessed 2019-11-11]
16. Cechetti NP. TEDE UPF. 2018. A Gamification Proposition on m-Health Systems for User's Engagement URL: http://tede.upf.br/jspui/handle/tede/1464 [accessed 2019-11-11]
17. Cechetti NP, Bellei EA, Biduski D, Rodriguez JP, Roman MK, de Marchi AC. Developing and implementing a gamification method to improve user engagement: a case study with an m-Health application for hypertension monitoring. Telemat Inform 2019 Aug;41:126-138. [doi: 10.1016/j.tele.2019.04.007]
18. Biduski D, Bellei EA, Rodriguez JP, Zaina LA, de Marchi AC. Assessing long-term user experience on a mobile health application through an in-app embedded conversation-based questionnaire. Comput Hum Behav 2020 Mar;104:106169. [doi: 10.1016/j.chb.2019.106169]

XSL•FO
RenderX

19. Debon R. TEDE UPF. 2019. Influence of using an m-Health application on the health conditions of hypertensive patients URL: http://tede.upf.br/jspui/handle/tede/1799 [accessed 2019-11-11]
20. Ferretto LR. TEDE UPF. 2018. A System for Recommending Physical Activities to Hypertensive Patients in the Context of e-Health URL: http://tede.upf.br/jspui/handle/tede/1465 [accessed 2019-11-11]
21. Zhou H, Lu Y, Chen W, Wu Z, Zou H, Krundel L, et al. Stimulating the comfort of textile electrodes in wearable neuromuscular electrical stimulation. Sensors 2015 Jul 16;15(7):17241-17257. [doi: 10.3390/s150717241]
22. Neumann CL, Menne J, Schettler V, Hagenah GC, Brockes C, Haller H, et al. Long-term effects of 3-month telemetric blood pressure intervention in patients with inadequately treated arterial hypertension. Telemed J E Health 2015;21(3):145-150. [doi: 10.1089/tmj.2014.0058]
23. Brucki SM, Nitrini R, Caramelli P, Bertolucci PH, Okamoto IH. Sugestões para o uso do mini-exame do estado mental no Brasil. Arq Neuropsiquiatr 2003 Sep;61(3B):777-781. [doi: 10.1590/S0004-282X2003000500014]
24. Alfonso LM, Vea HD, Ábalo JA. Validación del cuestionario MBG (Martín-Bayarre-Grau) para evaluar la adherencia terapéutica en hipertensión arterial. Rev Cuba Salud Pública 2008;34(1). [doi: 10.1590/s0864-34662008000100012]
25. Matta SR, Luiza VL, Azeredo TB. Adaptacao brasileira de questionario para avaliar adesao terapeutica em hipertensao arterial. Rev Saude Publica 2013 Jun;47(2):292-300. [doi: 10.1590/s0034-8910.2013047003463]
26. Ministério da Saúde do Brasil. Protocolos do Sistema de Vigilância Alimentar e Nutricional – SISVAN na assistência à saúde. Brasília, Brazil: Ministério da Saúde do Brasil; 2008.
27. O'Brien HL, Toms EG. The development and evaluation of a survey to measure user engagement. J Am Soc Inf Sci Technol 2010;61(1):50-69. [doi: 10.1002/asi.21229]
28. Davis FD, Bagozzi RP, Warshaw PR. User acceptance of computer technology: a comparison of two theoretical models. Manag Sci 1989 Aug;35(8):982-1003. [doi: 10.1287/mnsc.35.8.982]
29. Brooke J. SUS: a quick and dirty usability scale. In: Jordan PW, Thomas B, Weerdmeester BA, McClelland IL, editors. Usability Evaluation In Industry. First Edition. London, England: Taylor & Francis; 1996:189-194.
30. Gomes R, Deslandes SF. Social Research: Theory, Method and Creativity. Petrópolis, Brazil: Vozes; 2003.
31. Kim JY, Wineinger NE, Steinhubl SR. The influence of wireless self-monitoring program on the relationship between patient activation and health behaviors, medication adherence, and blood pressure levels in hypertensive patients: a substudy of a randomized controlled trial. J Med Internet Res 2016 Jun 22;18(6):e116 [FREE Full text] [doi: 10.2196/jmir.5429] [Medline: 27334418]
32. Piette JD, Datwani H, Gaudioso S, Foster SM, Westphal J, Perry W, et al. Hypertension management using mobile technology and home blood pressure monitoring: results of a randomized trial in two low/middle-income countries. Telemed J E Health 2012 Oct;18(8):613-620. [doi: 10.1089/tmj.2011.0271]
33. Lee CH, Chang BY. Effect of disease improvement with self-measurement compliance (measurement frequency level) in SmartCare hypertension management service. Telemed J E Health 2016 Mar;22(3):238-245. [doi: 10.1089/tmj.2015.0045]
34. Chauvin J, Perera Y, Clarke M. Digital technologies for population health and health equity gains: the perspective of public health associations. J Public Health Pol 2016 Nov 29;37(S2):232-248. [doi: 10.1057/s41271-016-0013-4]
35. Silveira DV, Marcolino MS, Machado EL, Ferreira CG, Alkmim MB, Resende ES, et al. Development and evaluation of a mobile decision support system for hypertension management in the primary care setting in Brazil: mixed-methods field study on usability, feasibility, and utility. JMIR Mhealth Uhealth 2019 Mar 25;7(3):e9869. [doi: 10.2196/mhealth.9869] [Medline: 30907740]
36. Hossain N, Yokota F, Sultana N, Ahmed A. Factors influencing rural end-users' acceptance of e-Health in developing countries: a study on portable health clinic in Bangladesh. Telemed J E Health 2019 Mar;25(3):221-229. [doi: 10.1089/tmj.2018.0039] [Medline: 29664328]
37. Jetty A, Moore MA, Coffman M, Petterson S, Bazemore A. Rural family physicians are twice as likely to use Telehealth as urban family physicians. Telemedicine and e-Health 2018 Apr;24(4):268-276. [doi: 10.1089/tmj.2017.0161] [Medline: 28805545]
38. Weiner JP. Doctor-patient communication in the e-health era. Isr J Health Policy Res 2012 Aug 28;1(1):33 [FREE Full text] [doi: 10.1186/2045-4015-1-33] [Medline: 22929000]

Abbreviations

eHealth: electronic health
MBG: Martín-Bayarre-Grau
SIS-Hiperdia: Brazilian Clinical Management System of Arterial Hypertension and Diabetes Mellitus
SISVAN: Dietary intake questionnaire by the Brazilian Food and Nutrition Surveillance System
SUS: System Usability Scale
TAM: technology acceptance model

XSL•FO
RenderX

Edited by G Eysenbach; submitted 28.08.19; peer-reviewed by E Burner, T Toro-Ramos; comments to author 03.10.19; revised version received 03.10.19; accepted 05.11.19; published 20.01.20

Please cite as:
De Marchi ACB, Alves ALSA, Gonçalves CBC, Cervi CR, Biduski D, Bellei EA, Madalozzo GA, Da Cruz IBM, Veiga JE, Rodriguez JPM, Ferretto LR, Bin LCP, Rebonatto MT, Portella MR, Roman MK, Cechetti NP, Rieder R, Debon R, Volpi SS
An Electronic Health Platform for Monitoring Health Conditions of Patients With Hypertension in the Brazilian Public Health System: Protocol for a Nonrandomized Controlled Trial
JMIR Res Protoc 2020;9(1):e15299
URL: http://www.researchprotocols.org/2020/1/e15299/
doi: 10.2196/15299
PMID:

©Ana Carolina Bertoletti De Marchi, Ana Luisa Sant' Anna Alves, Carla Beatrice Crivellaro Gonçalves, Cristiano Roberto Cervi, Daiana Biduski, Ericles Andrei Bellei, Guilherme Afonso Madalozzo, Ivana Beatrice Mânica Da Cruz, Jeangrei Emanoelli Veiga, João Pedro Mazuco Rodriguez, Luciano Rodrigo Ferretto, Luiz Carlos Pereira Bin, Marcelo Trindade Rebonatto, Marilene Rodrigues Portella, Mateus Klein Roman, Nathália Pinto Cechetti, Rafael Rieder, Raquel Debon, Simiane Salete Volpi. Originally published in JMIR Research Protocols (http://www.researchprotocols.org), 20.01.2020. This is an open-access article distributed under the terms of the Creative Commons Attribution License (https://creativecommons.org/licenses/by/4.0/), which permits unrestricted use, distribution, and reproduction in any medium, provided the original work, first published in JMIR Research Protocols, is properly cited. The complete bibliographic information, a link to the original publication on http://www.researchprotocols.org, as well as this copyright and license information must be included.

XSL•FO
RenderX
